# Development and Psychometric Properties of the Yoga Self-Efficacy Scale (YSES)

**DOI:** 10.1186/s12906-015-0981-0

**Published:** 2016-01-06

**Authors:** Gurjeet S. Birdee, Stephanie J. Sohl, Ken Wallston

**Affiliations:** 1 Department of Medicine, Vanderbilt University Medical Center, Nashville, Tennessee USA; 2 School of Nursing, Vanderbilt University Medical Center, Nashville, TN 37232-8300 USA

**Keywords:** Yoga, Self-efficacy, Health competence, Scale development, Health behavior

## Abstract

**Background:**

Yoga is a behavioral practice that uses physical movement, breathing, and meditation to improve health and promote personal transformation. Ancient yoga philosophy proposed that an individual’s confidence about yoga, a concept similar to self-efficacy, will affect the likelihood of improved health from yoga practice. The purpose of this study was to develop and examine the psychometric properties of a self-efficacy measure for yoga practice (the Yoga Self-Efficacy Scale; YSES).

**Methods:**

Yoga practitioners were recruited to evaluate the psychometric properties of YSES via a secure online survey. We collected data on additional measures to further examine construct validity. After two weeks, participants were invited to complete YSES items again to assess test-retest reliability.

**Results:**

A majority of participants (*N* = 309) were White (85 %), female (82 %), and yoga instructors (56 %). The 12-item YSES is unidimensional with a Cronbach’s alpha of 0.93. Test-retest reliability is *r* = 0.79 (*n* = 170). YSES scores are positively correlated with health competence, health-related quality of life, and years practicing yoga, supporting construct validity. Also, yoga teachers scored significantly higher on the YSES than non-teachers (*p* < 0.001). Non-significant relationships with education, income and sex supported discriminant validity. YSES maintained internal consistency and construct validity for all yoga styles surveyed.

**Conclusion:**

YSES is a reliable and valid measure of self-efficacy for yoga practice that may provide insight into barriers to adopting and maintaining yoga as a health behavior.

## Background

Yoga is a behavioral practice that uses physical movement, breathing, and meditation to improve health and promote personal transformation [1]. Presently, 20 million adults in the United States practice yoga for health promotion [2]. About 2000 years ago, Patanjali wrote the Yoga Sutra describing the process and goals of yoga. In this seminal Indian yoga text, he writes that confidence will provide the energy to achieve the goals of yoga against all odds and higher confidence will achieve these goals faster (Yoga Sutra 1.20-1.21 translated by TKV Desikachar) [1]. Patanjali also proposed that an individual’s confidence in his or her yoga practice affects the likelihood of improved physical and mental health. This ancient premise parallels the modern construct of self-efficacy in behavioral science.

The word in Sanskrit for confidence is śraddha [3]. One synonym in psychology for confidence is self-efficacy. Self-efficacy is the confidence that one is able to perform goal-directed behaviors [4] or perceived competence for performing those behaviors [5]. Self-efficacy influences health behavior choices and effort, and relates to health outcomes [6]. Measures of health self-efficacy vary in their level of specificity. Some measures assess perceived competence for general health across situations [7, 8] while others assess competence for a particular health behavior such as exercise in the context of specific potential barriers [9]. Self-efficacy measures may also vary as an individual considers adopting or commits to maintaining a health behavior [10].

Research on yoga practice and self-efficacy is sparse [11–13]. The only self-efficacy measure specific to yoga practice was an adapted shortened version of Bandura’s Exercise Self-Efficacy Scale [13, 14]. This measure identified perceived competence for practicing yoga when challenged with specific barriers (such as being too tired or in a bad mood) related to yoga class attendance and home practice. The psychometric properties of this measure were not reported and the list of potential barriers assessed was not exhaustive. No study, to our knowledge, has investigated the association between yoga self-efficacy and health outcomes.

The three main tools of yoga are body, breath, and mind. While various styles of yoga exist, one popular and influential style is yoga in the Krishnamacharya tradition (or Viniyoga). Basic descriptions of each yoga tool according to Krishnamacharya [1] are:

- ➢ Body: Physical movements are coupled to breathing requiring practitioners to dual-task, which demands increased cognitive attention.
- ➢ Breath: Breathing exercises may also be performed without physical movement (called *pranayama).* Usually breathing is done deeply and slowly with experienced practitioners reducing respiratory rates substantially below resting (less than 6 breaths a minute).
- ➢ Mind: Meditation is the practice of focusing the mind in a single direction. The direction or object of focus varies based on the goal of the meditation practice. Objects of meditation may be within the body (e.g., breathing, thoughts, and body parts) or mind (e.g., thoughts or feelings) or external (e.g., images of nature or religious deities).

While practicing, yoga emphasizes three main qualities: focus of the mind; ease or gentleness (Sanskrit-sukham); and strength or steadiness (Sanskrit-sthira). The ability to focus the mind or attention is a skill that develops gradually over time from regular yoga practice. Physical movements of the body and breathing exercises are used to train the mind to focus and prepare for meditation. Yoga also encourages individuals to practice with ease without physical, breath, or mental strain. Further, yoga instructs individuals to practice with strength and steadiness in regards to movement, breath, and meditation. Yoga requires that each exercise encompass the dual qualities of ease and strength in body, breath and mind while maintaining focus. Yoga theory maintains that a yoga practice with these three qualities will be more effective for the individual than if only one or two of the qualities were incorporated.

The purpose of this study was to develop and examine the psychometric properties of a new self-efficacy measure for yoga practice (the Yoga Self-Efficacy Scale--YSES). The YSES is designed to inform research on the clinical application and mechanisms of yoga as a health behavioral intervention. A self-efficacy measure specific to yoga may provide insight into barriers to adopting and maintaining yoga as a health behavior. Also, as with other self-efficacy measures, YSES may correlate with health outcomes as yoga is applied clinically. Yoga is represented by many different styles and traditions. We focused on yoga from the Krishnamacharya tradition (Viniyoga), as this is the yoga style we study our clinical research. This instrument was developed based on the fundamental tools and qualities perceived as competencies of a yoga practitioner according to the Krishnamacharya tradition. For the purposes of this study we designate Krishnamacharya yoga as the target yoga, but we also investigate the psychometric properties of the YSES when administered to practitioners of other yoga styles.

We developed the following hypotheses based on our experience teaching yoga, yoga theory, and self-efficacy theory:

1. As evidence for concurrent validity, YSES scores would correlate positively with health competence.
2. As evidence for convergent validity, YSES scores would correlate positively with health-related quality-of-life.
3. As evidence for the new scale’s discriminant validity, YSES scores would be uncorrelated with social desirability bias, sex, and indicators of socioeconomic status (i.e., education and income).
4. As evidence for the new scale’s known-groups construct validity, participants who have practiced yoga for longer periods, were older, yoga teachers, or reported more frequent practice would have higher YSES scores.
5. We also hypothesized that there would be insignificant differences between the reliability and validity of the YSES between participants who practice the target yoga and those who practice other forms of yoga.

## Method

### Procedure for item pool generation and development of YSES

This study followed a systematic procedure recommended for scale development [15, 16]. The authors generated an original list of 21 yoga self-efficacy items based on yoga [1] and self-efficacy theory [4, 10]. We generated items that measure an individual’s perceived competence to maintain the three fundamental qualities of yoga (focus, ease, and strength) across situations, rather than in relation to specific barriers. *A priori*, we categorized items into three factors reflecting the three tools of yoga: body, breath, and mind. Items were rated on a 9-point Likert scale ranging from *strongly disagree* to *strongly agree.*

To insure content validity, the original items were expanded and refined through a process of expert consensus. The expert panel consisted of three yoga teachers/therapists and the principal investigator, Dr. Gurjeet Birdee (GB), who is also an expert yoga therapist. All three yoga experts were advanced teachers with more than 50 collective years teaching yoga from the target yoga in the Krishnamacharya tradition. We designed the instrument based on this tradition to enhance the clarity of operationalizing yoga due to the wide variety of styles. The experts were consulted to review and refine original items and add new items. The resulting list consisted of 32 items. Common themes identified by experts were then reworded in the form of an expectancy or belief regarding confidence in one’s own ability to achieve each behavior and repetitive or redundant items were removed. The expert panel along with the researchers refined the list to 14 self-efficacy items.

Next, we performed cognitive interviews with yoga teachers who taught and practiced the target yoga regularly. Two researchers were present for each cognitive interview. One researcher asked questions while the other researcher primarily took notes. Cognitive interviews were audio recorded to enable clarification, as needed. Interviews were performed in two rounds. First, we interviewed three yoga teachers. After reviewing the data and feedback from these three interviews we revised the item list. We then performed additional cognitive interviews with three other yoga teachers based on the revised item list. These semi-structured interviews used verbal probes following volunteers’ responses to each item to evaluate comprehension, what information they used to respond to the items, how they came to a decision regarding the responses they gave to the items, and ease of ability to respond with the options provided.[17] The items were further revised or deleted based on feedback from notes taken during the second set of cognitive interviews. An effort was made to write items simply and directly. The revised item pool consisted of 13 items with a Flesch-Kincaid Readability Grade-level of 5.3. The final item list is shown in Table 1.

**Table 1:** Yoga Self-Efficacy Scale Items

Yoga tool	Item
Body	When I practice yoga…
	1. I am able to remain as comfortable as possible while doing movements.
	2. I am able to keep my mind focused on movements of my body.
	3. I can coordinate the movements of my body with my breath.
	4. I am able to move my body smoothly.
	5. I am able to maintain a feeling of stability in my body.
Breath	When I practice yoga…
	6. I am able to keep my breath smooth and continuous.
	7. I am able to remain comfortable while regulating my breath.
	8. I am able to focus my mind on my breath.
	9. I am able to make my breath longer and deeper without feeling anxious.
Mind	During my yoga practice…
	10. If distracted, I can re-focus my mind.
	11. (I am confident that I can maintain my attention.)*
	12. If asked, I am able to visualize or have an impression of an object in my mind.
	13. I am able to remain focused on a meditative object or point.

*Removed after psychometric analysis

Note: All items were rated on a 9-point Likert response scale ranging from 1 = “strongly disagree” to 9 = “strongly agree”

### Evaluation of the yoga self-efficacy scale

#### Participants

We only recruited participants who practiced yoga to participate in the study because the YSES was designed for individuals who have been exposed to yoga. Therefore, non-yoga practitioners would not be able to respond to the items. We targeted recruitment efforts to practitioners of Krishnamacharya or Viniyoga (the target yoga), but also included any other yoga practitioner (non-target yoga). We obtained email addresses through personal contacts of the principal investigator (GB) and publicly available online lists from national yoga associations (American Viniyoga Institute). All study procedures were approved by the Vanderbilt University Institutional Review Board.

#### Enrollment

Individuals were sent an email describing the research study and asked to click on a link to a website with the survey. The email described the study as developing a new instrument for yoga research and the estimated time required for participation, but did not specifically describe any aims or measures. Individuals were encouraged to forward the email to yoga students and yoga colleagues to invite them to participate. Individuals who visited the website were presented an online informed consent document. Informed consent was obtained from all individual participants included in the study.

### Data collection

Those who consented to participate in the study were asked to complete baseline data collection. We used Research Electronic Data Capture (REDCap), an online research management tool developed at Vanderbilt, for administering the research survey, collecting data, and monitoring participation [18]. REDCap is a secure, web-based application designed exclusively to support data capture for research studies. After two weeks, participants who completed the baseline data collection were invited by email to complete the 13 YSES items again to assess test-retest reliability. Other baseline survey instruments were not repeated. Email addresses were kept confidential. *Other Measures.*

We collected data at baseline on health self-efficacy, health-related quality of life, social desirability bias, yoga practice characteristics, and sociodemographics.

#### Health competence

The 8-item Perceived Health Competence Scale [7] was used to assess health self-efficacy, the degree to which an individual feels capable of effectively managing his or her health behavior [5]. A sample item from this instrument is, “It is difficult for me to find effective solutions for health problems that come my way.” This scale has high internal consistency (Cronbach’s alpha range: 0.82 - 0.90) and construct validity in healthy populations and persons with chronic illness. In our study this instrument had a Cronbach’s alpha of 0.86.

#### Health-related quality of life

The 10-item PROMIS Global Health scale was implemented to assess health-related quality of life with both a physical and mental health component [19]. A sample item from this instrument is, “In general, would you say your health is [poor, fair, good, very good, or excellent]?” This instrument has good internal consistency, construct validity, and responsiveness and has been shown to be precise and reliable in the general adult U.S. population [20, 21]. In our study this instrument had a Cronbach’s alpha of 0.86.

#### Social desirability bias

*A* 5-item version of the Marlowe-Crown Social Desirability Scale was used to assess social desirability bias. This short version has been shown to be valid and reliable [22]. A sample item from this instrument is, “I am always courteous even to people who are disagreeable.”

#### Yoga practice characteristics

We asked what type(s) or style(s) of yoga the participant practiced most often (*Iyengar, Ashtanga, Bikram, Power, Kundalini, Krishnamacharaya or Viniyoga, Sivananda, Kripalu, Anusara, Hatha, or other [specify type]*). Participants were asked about the number of years they have practiced yoga (*less than 3 years, more than 3 years*), frequency of practice a week (*1*–*7 days*), and average practice length (*in minutes*). They were also asked if they were a certified yoga teacher (Yes/No).

#### Sociodemographics

We obtained self-reported information on age, sex, ethnicity, race, education, and income.

### Analyses

All analyses were conducted using IBM SPSS Statistics Version 22 and the Amos add-on (Armonk, New York). Corrected item-total correlations and association of the YSES item scores with social desirability scores were reviewed to evaluate and optimize scale performance. Cronbach’s alpha was calculated for the YSES scale to estimate internal consistency reliability. Test-retest reliability was evaluated using a series of paired t-tests to determine test-retest correlations and mean item scores changes over the 2-week interval between test administrations. Concurrent, convergent, and discriminant validity were assessed using independent groups t-tests and Spearman's correlations (chosen due to the skewed nature of the total scale). Comparisons of correlations between practitioners of the target yoga group and those practicing other yoga styles were evaluated by a z-test.

A confirmatory factor analysis was conducted using structural equation modeling to test the *a priori* determined structure of one higher order latent factor composed of three separate, but highly correlated latent factors --Body (item #s 1, 3, 4, & 5), Breath (item #s 6, 7, 9) and Mind (items #s 2, 8, 10, 11, 12, & 13; Table 2). A significant Bollen-Stine bootstrap (a modified method of the χ^2^ statistic for a non-normal sample) indicated if a model was not a good fit, although this statistic is highly sensitive to sample size. Thus, other indicators of model fit considered were the RMSEA with a target value of < 0.08 and CFI with a target value of > 0.95.[23] Due to the non-normal sample, standardized bootstrapped-corrected estimates were checked to determine significance of paths in the model.

**Table 2:** Sample characteristics (*n* = 309)

Characteristic	
Age (mean, S.D.)	51 (13)
Female (n, %)	253 (82)
Race (n, %)	
White	262 (85)
Black	7 (2)
Asian	15 (5)
Some other race or missing	25 (8)
Hispanic, Latino or Spanish in origin (n, %)	15 (5)
Annual income (U.S. dollars) (n, %)	
Less than $24,999	34 (12)
25000-44999	50 (16)
50000-74999	48 (16)
75000-99999	46 (15)
100000 or more	78 (25)
Decline to answer	35 (11)
Education (n, %)	
High school graduate/GED/ or less	6 (2)
Vocational/technical school	8 (3)
Associate degree/some college	31 (10)
Bachelor’s degree	88 (29)
Advanced degree	158 (51)
Most frequent yoga style practiced (n, %)	
Krishnamacharya or Viniyoga (target yoga) (%)	159 (52)
Hatha	53 (17)
Anusara	8 (3)
Ashtanga	9 (3)
Kripalu	4 (1)
Bikram	1 (<1)
Kundalini	1 (<1)
Other	31 (10)
Certified yoga teacher (n, %)	174 (56)
On average, how many times a week do you practice yoga? (mean, S.D.)	5 (2)
When you practice yoga, on average how many minutes? (mean, S.D.)	54 (20)
How long have you been practicing yoga? (n, %)	
More than 3 years	53 (80)
3 years or less	246 (17)
Health Self-Efficacy^a^ (mean, S.D.)	32.96 (4.52)
Health-related Quality of Life^b^ (mean, S.D.)	3.93 (0.53)
Social Desirability Bias^c^ (mean, S.D.)	1.17 (1.28)

^a^Possible range: 8 – 40

^b^Possible range: 1 – 5

^c^Possible range: 0–5

## Results

### Sample characteristics

We administered the survey online to yoga practitioners (*N* = 309) to collect data for psychometric analysis. Table 3 describes participants’ sample characteristics. The participants had a mean age of 51 years (SD = 13.3) and were primarily white (85 %) and female (82 %). Approximately half of the participants (52 %) practiced the target yoga, and 56 % were certified yoga instructors.

**Table 3:** Construct validity correlations for the total sample and for those practicing the target yoga style vs. other yoga styles.

	YSES					
	Total Sample		Target Yoga		Other Yoga	
	n	rho	n	rho	n	rho
Health Self-Efficacy	293	0.39***	159	0.41***	134	0.32***
Health-related Quality of Life	291	0.34***	158	0.40***	133	0.24**
Social Desirability Bias	302	0.25***	159	0.32***	143	0.17*
Age	291	0.12*	158	−0.09	133	0.24**
Education	291	0.06	158	0.09	133	0.05
Income	256	0.03	139	0.22	117	0.10
Years of yoga practice	299	0.25***	159	0.25**	140	0.22**

^*^ *p* < 0.05, **p < 0.01,***p < 0.001

### Internal consistency, scale optimization, and test-retest reliability

Internal consistency for the initial 13-item YSES scale was very high (Cronbach’s alpha = 0.93), suggesting possible unnecessary redundancy. Examination of corrected item-total correlations for those items that were perceived by the authors as conceptually redundant (i.e., items 10, 11, 12, & 13) along with correlations between those items and social desirability scores resulted in the removal of item #11 (“I am confident that I can maintain my attention”). The Cronbach’s alpha for the resulting 12-item YSES was also 0.93 and the correlation of the YSES total score with social desirability was not changed. No further modifications were made in order to maintain the content validity of the YSES. Cronbach’s alpha for those who practice the target yoga is 0.93, while it is 0.91 for those who practice a different yoga style.

Test-retest reliability over 2-weeks for the 12-item YSES was *rho* = 0.79 (*n* = 170). YSES scores across time were stable (YSES total [SD] at baseline was 91.25 [11.74]; total [SD] at 2 weeks was 91.10 [10.60]; *p* = 0.80. Test-retest reliability was also similar by yoga type: target yoga (*n* = 100) *rho* = 0.76; other yoga styles (*n* = 70) *rho* = 0.75.

### Descriptive statistics

The total scores for the final 12-item version of the YSES ranged from 28 to 108 with a mean of 90.77, a median of 91, and a standard deviation of 11.62. The total YSES scores were negatively skewed, with a ratio of skewness to its standard error of −8.13. The kurtosis ratio was 13.83.

### Construct validity

In the second column in Table 3 we present correlational results related to construct validity for hypothesized relationships between the YSES and measures of health competence, health-related quality of life, social desirability bias, sociodemographic variables, and number of years of yoga practice for the sample as a whole. YSES scores were uncorrelated with education and income. YSES scores were significantly, positively correlated with health competence and health-related quality of life, and also with social desirability bias, number of years practicing yoga, and age, although this latter correlation was very weak. Those respondents who have practiced yoga for three years or less (*n* = 53) scored significantly lower on the YSES (*M* = 85.0) than those who have practiced for more than three years [*n* = 246; *M* = 91.9; *p* < 0.001].

In addition, we compared the correlations of the YSES and the psychosocial measures between those who practiced the target yoga style (Krishnamacharya or Viniyoga) versus those who practiced a different style of yoga (see columns four and six of Table 3). With one exception, correlational results related to construct validity were not significantly different when analyzed by yoga type. The one exception was the relationship between YSES scores and age. That correlation was negative and not significant for those practicing the target yoga style, but was significant and positive for those practicing other yoga styles (*z* = −2.82; *p* < 0.005).

Table 4 presents the results of t-tests comparing mean scores on the YSES and other psychosocial measures of those who practice the target yoga style vs. those who practice other styles of yoga. Although the two groups did not differ on social desirability bias, practitioners of the target yoga style scored significantly higher on yoga self-efficacy, health competence, and health-related quality of life than did other yoga practitioners.

**Table 4:** Differences in mean scores on psychosocial measures by yoga style

	Yoga style	N	Mean	S.D.	*t*-value (*p*)
YSES Total Score	Target	159	93.63	11.08	
	Non-Target	143	87.60	11.41	4.63 (<0.001)
Health Self-Efficacy	Target	159	33.72	4.12	
	Non-Target	134	32.04	4.81	3.22 ( 0.001)
Health-related Quality of Life	Target	159	20.12	3.04	
	Non-Target	133	19.19	3.49	2.43 (0.016)
Social Desirability Bias	Target	159	1.23	1.27	
	Non-Target	150	1.11	1.29	0.87 (0.387)

In further analyses, t-tests revealed that certified yoga teachers scored significantly higher (mean [SD]) on the YSES than those who were not teachers (yoga teachers 93.61 [10.40] vs. non-yoga teachers 86.68 [12.17]; *t* = 5.26, *p* < 0.001). In addition, we analyzed the relationship of the YSES with frequency of yoga practice by categorizing respondents into those who reported practicing yoga 3 days/week or less, 4 or 5 days/week, or 6 or 7 days/week. Respondents who indicated that they only practice yoga 3 days/week or less (*n *= 102) scored significantly lower on the YSES (86.04) than either of the other two groups (92.92 and 93.38, respectively; *F* = 13.48; *p* < 0.001) who did not differ from one another (*p* = 0.45). Male respondents did not differ from female respondents in total YSES scores (*p* = 0.98).

### Confirmatory factor analysis

**Fig. 1:**
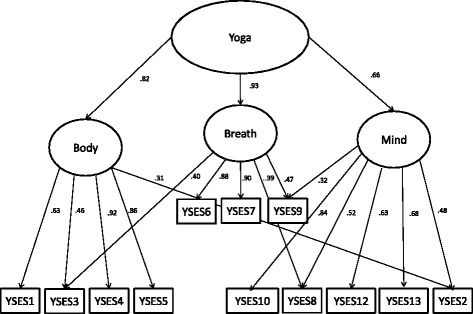
Exploratory confirmatory factor analysis of Yoga Self-Efficacy Scale (YSES) items

The confirmatory factor analysis of a model with three separate latent factors under a higher order latent yoga factor was not a good fit to the data (Bollen-Stine bootstrap *p* = 0.002; RMSEA = 0.11; CFI = 0.92). Therefore, an exploratory confirmatory factor analysis was then conducted to clarify the factor structure. Theoretically sound modification indices were applied incrementally with χ^2^ analyses indicating significant improvements at each step to achieve a model that was a good fit to the data [23]. This resulting model, shown in Fig. 1, also included paths from both the Body and Mind latent factors to item 2 (“I am able to keep my mind focused on movements of my body”), the Body and Breath latent factors to item 3 (“I can coordinate the movements of my body with my breath”), and the Breath and Mind latent factors to items 8 and 9 (“I am able to focus my mind on my breath;” “I am able to make my breath longer and deeper without feeling anxious”). The Bollen-Stine bootstrap for the resulting model was not significant (*p* = 0.104), indicating that the model was correct. In addition, the RMSEA (0.057) and CFI (0.981) model fit indices were within a desirable range[23].

## Discussion

Our analyses provide preliminary evidence supporting that the 12-item YSES is a reliable and valid measure for assessing self-efficacy for yoga practice. Specifically, we have shown that the YSES is both internally consistent and stable over time, and our hypothesized relationships for the construct validity of the YSES were confirmed. YSES scores correlated positively with health competence as evidence for concurrent validity, while non-significant correlations with education and income provide evidence for the YSES’s discriminant validity. The positive correlation of the YSES with health-related quality-of-life demonstrates convergent validity. Participants who were older, certified yoga teachers, practiced for more years or frequently had higher YSES scores. These latter results are evidence of known-groups construct validity. In addition, YSES maintained internal consistency, test-retest reliability and construct validity for both target and non-target yoga groups, supporting that this instrument may be used regardless of yoga style. The applicability of the YSES to other yoga traditions may be because the target yoga practices are closely tied to the Yoga Sutra, which serves as a foundational text for the teachings of most yoga traditions.

Analyses exploring whether validity of YSES differed by type of yoga practiced revealed that YSES scores were higher among the target group than the non-target group. One reasonable explanation for this result is that yoga practitioners who were specifically instructed in the target yoga techniques assessed by the YSES had a higher perceived competence for performing these techniques. Future research may also examine whether those who have more than a token experience with yoga differ from novice practitioners in the relationship between YSES scores and the likelihood of commitment to regular yoga practice [10]. Target yoga practitioners also had significantly higher scores on health competence. Theoretically, successful behavioral changes specific to practicing yoga may also contribute to a more general sense of competency [24]. Further, target yoga practitioners scored higher on health-related quality of life than non-target practitioners. Without further research, the implications of higher quality of life in this cross-sectional sample of practitioners of Krishnamacharya yoga are unknown. We were also able to distinguish differences in the YSES among yoga teachers versus non-yoga teachers suggesting that increased training and experience produces higher yoga self-efficacy.

Factor analyses clarified that the YSES is most simply conceived of as a unidimensional measure because it is necessary for some items to load on more than one sub-factor. This result is consistent with yoga philosophy and practice [1]. One of the fundamental goals of yoga is to increase cognitive attention. To help focus the mind, practitioners are asked to precisely coordinate body movements and breathing. For example the yoga instruction, “Raise your arms overhead while you inhale, then lower your arms while you exhale,” requires cognitive attention as arm movement is coordinated with breath. Since Krishnamacharya yoga performs all movements coordinated with breath, a logical result is that items assessing body and breath techniques would load on more than one factor. Similarly, body movements and breathing are performed with specific mind techniques, so items that assess movement and mind or breathing and mind would also load on more than one factor. There are yoga practices in which no movement is performed such as pranayama (breathing only) and dhyana (meditation). We did not analyze such yoga practices separately for this study. Thus, YSES presently is intended for use in a population that is using the full range of yoga techniques rather than one in isolation. Further, based on results and the interconnected components of yoga (i.e., body, breath, and mind), we recommend use of the entire scale at this time.

### Limitations

Despite our evidence that the YSES is a valid tool, our study has limitations. We utilized a specific yoga type, Krishnamacharya yoga, to frame yoga self-efficacy. Other yoga traditions may define self-efficacy or mastery differently based on varying techniques and philosophy. However, a strength of our study is that the YSES worked well (i.e., was reliable and valid) even when completed by practitioners trained in other styles of yoga. In addition, our sample consisted mostly of white, female, educated women who had substantial experience with yoga practice. Thus, this sample may not have enough variability to examine how YSES operates among men, novice yoga practitioners, or a more diverse racial and socioeconomic group. In addition, there may be some bias toward providing desirable responses on the YSES introduced by factors such as experience, social desirability, or positively-worded items. Older or long-term yoga practitioners may have reported higher competence based on the belief that longer practice relates to advance skill; however, the significant association of the YSES with social desirability bias was relatively weak. This significant association of YSES with social desirability bias could be partially due to yoga philosophy and practice which might enhance socially desirable behavior among yoga practitioners and thus result in higher ratings on the measure of social desirability (e.g., “I am always courteous even to people who are disagreeable”) than are typically considered desirable. Furthermore, as a cross-sectional study, we are unable to identify causal relationships between YSES and other factors.

## Conclusion

In summary, the YSES is a reliable and valid instrument to measure self-efficacy for yoga practice. This tool may be useful for epidemiological and clinical studies of how yoga practice is related to health. For example, higher yoga self-efficacy may correlate with increased adherence and maintenance to yoga practice. The YSES may also be useful to understand why some individuals benefit more from yoga practice than others. Specifically, higher YSES scores may correlate with health outcomes in clinical research. Improvements in general measures of self-efficacy have been shown to mediate changes in clinical yoga studies of back pain [11] and psychological health [12]. Future research might investigate the longitudinal relationship between YSES and adherence to yoga practice and health outcomes. Such studies would provide evidence for Pantanjali’s two thousand year old hypothesis that individuals’ confidence in their yoga practice predicts benefits.
